# Association of Human Papilloma Virus, Cytomegalovirus, and Epstein–Barr Virus with Breast Cancer in Jordanian Women

**DOI:** 10.3390/medicina60050699

**Published:** 2024-04-25

**Authors:** Ashraf I. Khasawneh, Nisreen Himsawi, Ashraf Sammour, Sofian Al Shboul, Mohammed Alorjani, Hadeel Al-Momani, Uruk Shahin, Hafez Al-Momani, Moureq R. Alotaibi, Tareq Saleh

**Affiliations:** 1 Department of Microbiology, Pathology, and Forensic Medicine, Faculty of Medicine, The Hashemite University, Zarqa 13133, Jordanhafez@hu.edu.jo (H.A.-M.); 2 Department of Anatomy, Physiology & Biochemistry, Faculty of Medicine, The Hashemite University, Zarqa 13133, Jordan; 3 Department of Pharmacology and Public Health, Faculty of Medicine, The Hashemite University, Zarqa 13133, Jordan; 4 Department of Pathology and Microbiology, Faculty of Medicine, Jordan University of Science and Technology, Irbid 22110, Jordan; 5 Department of Pharmacology and Toxicology, College of Pharmacy, King Saud University, Riyadh 11421, Saudi Arabia

**Keywords:** HPV, CMV, EBV, breast cancer, RT-PCR, Jordan

## Abstract

*Background and Objectives*: The investigation of oncogenic viruses and their potential association with breast cancer (BC) remains an intriguing area of study. The current work aims to assess evidence of three specific viruses, human papillomavirus (HPV), cytomegalovirus (CMV), and Epstein–Barr virus (EBV) in BC samples and to explore their relationship with relevant clinicopathological variables. *Materials and Methods*: The analysis involved BC samples from 110 Jordanian female patients diagnosed with BC and breast tissue samples from 30 control patients with no evidence of breast malignancy, investigated using real-time PCR. The findings were then correlated with various clinico-pathological characteristics of BC. *Results*: HPV was detected in 27 (24.5%), CMV in 15 (13.6%), and EBV in 18 (16.4%) BC patients. None of the control samples was positive for HPV or CMV while EBV was detected in only one (3.3%) sample. While (HPV/EBV), (HPV/CMV), and (EBV/CMV) co-infections were reported in 1.8%, 2.7%, and 5.5%, respectively, coinfection with the three viruses (HPV/CMV/EBV) was not reported in our cohort. A statistically significant association was observed between HPV status and age (*p* = 0.047), and between clinical stage and CMV infection (*p* = 0.015). *Conclusions*: Our findings indicate the presence or co-presence of HPV, CMV, and EBV in the BC subpopulation, suggesting a potential role in its development and/or progression. Further investigation is required to elucidate the underlying mechanisms that account for the exact role of oncoviruses in breast carcinogenesis.

## 1. Introduction

According to the World Health Organization (WHO), breast cancer (BC) was the most common cancer on the global level and the leading cause of female cancer deaths in 2020 [1]. In the Arab world, Jordan is among the top ten countries in BC incidence and death rates [2], with BC being the most diagnosed female malignancy (38.4%) [3]. Subsequently, a thorough understanding of BC’s etiology, molecular pathways, risk factors, and health outcomes is essential.

The development of BC is multifactorial, with a set of proposed contributing elements that can increase its risk including age, smoking, alcohol intake, genetic predisposition, and environmental factors, particularly viral infections [4]. It is hypothesized that several types of viruses can infect breast epithelial cells and become dormant, causing what is called a “latent infection”. These types of infections contribute to cancer development through multiple molecular pathways. For example, a virus can inhibit apoptosis in the infected cell [5], it can integrate its DNA in an oncogenic region of the cell’s DNA [6], activate telomerase, which contributes to cellular immortality [7], or induce mitosis in fully differentiated cells leading to their uncontrollable division [8]. Moreover, the effect of many oncogenic viruses is propagated through affecting anti-oncogenic proteins and interfering with the function of cell cycle regulators such as p53, RAS, retinoblastoma protein (Rb), and many others [8,9]. In addition, viruses can contribute to cancer development through invading the immune system and interfering with the elimination of viral particles and virus-infected cells [10,11].

Several viruses fall into this category including the Epstein–Barr virus (EBV) [12], the human papilloma virus (HPV) [13], the cytomegalovirus (CMV) [14], and others [15,16]. These viruses are linked to various cancers, including neuroblastoma, B cell lymphomas, and cervical and oropharyngeal cancers [17,18,19,20]. The association between being infected with these viruses and the occurrence of BC is still a matter of active research with no conclusive evidence. Worldwide, chronic infections (both viral and bacterial) are the cause of a fifth of cancer cases, and in developing countries, they are responsible for 20% of cancer-related deaths [21]. Several studies in our region showed a positive correlation between viral infections and BC in Iran, Pakistan, Syria, and Sudan [22,23,24,25], while other studies have rejected this causal relationship [26,27], indicating that a role for viral infections and the pathogenesis of BC is still unresolved.

In Jordan, only a single published study has evaluated the association between BC and viral infections such as EBV and HPV [28]. The aim of this study was to further investigate evidence of HPV, EBV, and CMV in BC samples obtained from a Jordanian female subpopulation.

## 2. Materials and Methods

### 2.1. Sample Collection

A retrospective screening of the medical records of King Abdullah University Hospital (KAUH) patients, diagnosed with BC between 2018 and 2022, was performed. The eligibility criteria for patient inclusion were as follows: patients with a histologically confirmed diagnosis of BC, aged between 20–80 years, who had not undergone radiotherapy or chemotherapy prior to tumor excision, and their samples tested positive for GAPDH (housekeeping gene) by PCR.

The samples comprised 120 formalin-fixed paraffin-embedded (FFPE) tissue blocks of BC along with 30 samples of non-malignant breast lesions as controls. Of those 120 BC samples, 10 samples were excluded from the analysis due to the unavailability of their molecular subtyping data. The samples were obtained from KAUH, Irbid, Jordan. All research activities within this project were carried out in accordance with ethical approval granted by the Hashemite University Institutional Review Board (IRB) (No. 7/14/2020/2021) and KAUH IRB (No. 42/151/2022).

### 2.2. DNA Extraction

FFPE breast tissue blocks were obtained from the KAUH Pathology Department for each patient. A 20 μm thick section was prepared from each block, and genomic DNA extraction was performed using the QIAamp DNA FFPE Tissue Kit (Qiagen, Hilden, Germany) following the manufacturer’s protocol. Briefly, the FFPE samples were deparaffinized through sequential washes with warmed (100%) xylene and (100%) ethanol to remove residual xylene. Following this, the samples were lysed using 20 μL proteinase K and ATL lysis buffer (Roche, Munich, Germany) and incubated for two hours at 56 °C initially, followed by an additional hour at 90 °C to reverse formalin crosslinking. Subsequently, 4 μL of RNAse (100 mg/mL) was added, and the mixture was incubated at room temperature for 2 min. Next, AL buffer and ethanol were added, mixed thoroughly, and the lysate containing DNA was transferred to the MinElute column for several washing steps. Finally, DNA was eluted in 200 μL ATE buffer and stored at −20 °C for subsequent experiments. The concentration of DNA was measured using a Qubit 3.0 Fluorometer (Thermo Fisher Scientific, Waltham, MA, USA) following the manufacturer’s instructions.

### 2.3. HPV Detection in Breast Cancer Samples

HPV detection and genotyping were conducted using the REALQUALITY RQ-Multi HPV detection kit (AB ANALITICA, Padua, Italy), as previously described [17]. This kit enables the identification of 28 HPV genotypes, including 14 high-risk (16, 18, 31, 33, 35, 39, 45, 51, 52, 56, 58, 59, 66, 68), 6 potential high-risk (26, 53, 67, 70, 73, 82), and 8 low-risk (6, 11, 40, 42, 43, 44, 55, 83) types. It amplifies a short sequence within the E6 and E7 genes of the HPV genome, individually detecting HPV 16 and HPV 18 genotypes, while other genotypes are detected as pools. Additionally, human beta-globin serves as an internal control. The kit includes both negative and positive controls, with the positive control containing plasmid DNA fragments of HPV 16, 18, and 33 for high-risk HPV, and fragments of HPV 6, 26, and 40 genotypes for low-risk HPV.

RT-PCR amplification was conducted using a Bioer RT-PCR thermocycler (China). The PCR mixture had a total volume of 25 μL, consisting of 22.5 μL of master mix and 2.5 μL of template DNA (3–15 ng/µL), positive control, or negative control. Thermocycling conditions included an initial step of 2 min at 50 °C for UNG activation, followed by initial denaturation at 95 °C for 10 min, then 45 cycles at 95 °C for 15 s, 60 °C for 60 s, and 72 °C for 60 s. Data were interpreted according to the manufacturers’ guidelines. Briefly, for HPV-negative samples, an amplification signal of the internal control (beta globin) with a Ct value ≤ 34 was regarded as appropriate, while values exceeding 34 were considered inadequate. Conversely, internal control values exceeding 34 were considered acceptable for HPV-positive samples, as per the manufacturer’s guidelines. Regarding viral target detection, any amplification signal demonstrating a proper sigmoid curve shape with a Ct value ≤ 40 was classified as positive.

### 2.4. EBV and CMV Detection in Breast Cancer Samples

To initially assess the quality of the extracted DNA samples, all samples underwent PCR using GAPDH primers. The GAPDH primers used were as follows: forward primer (GAPDH-F): 5′ GAGTCAACGGATTTGGTCGT, reverse primer (GAPDH-R): 5′ TTGATTTTGGAGGGATCTCG, generating a PCR product of 237 bp. Additionally, for the detection of human cytomegalovirus (HCMV), primers conserved for the GB region were utilized. The forward primer sequence was 5′-TCTGGGAAGCCTCGGAACG, and the reverse primer sequence was 5′-GAAACGCGCGGCAATCGG. To screen BC samples for the prevalence of the EBV-EBNA 2 gene, specific primers EBNA2F: 5′-AGGACAGCCGTTGCCCTAGTG and EBNA2R: 5′-TAGCGGACAAGCCGAATACCCT targeting the EBV-EBNA2 gene were utilized.

Initially, a conventional PCR was conducted to determine the optimal annealing temperature for each primer. Subsequently, the QuantiTect SYBR^®^ Green PCR Kit (Cat. No. 204143, QIAGEN) was employed to amplify the target DNA for each sample using the aforementioned primers. In the conventional PCR protocol, each reaction included 12.5 µL of Taq DNA polymerase, 2 µL of template DNA, 8.5 µL of nuclease-free water, and 1 µL each for the forward and reverse primers per sample. Positive and negative controls were included in every run. The resulting PCR products were then loaded onto a 2% agarose gel, and the DNA bands were visualized and documented using a gel documentation system. After completion of the run, a UV light device was utilized to visualize the distinct DNA bands.

During the reaction setup, both the samples and all reagents from the QuantiTect SYBR^®^ Green PCR Kit were kept on ice. Using filter tips, 12.5 μL of QuantiTect SYBR Green PCR Master Mix, 8.5 μL of RNase-free water, 2 µL of template DNA, and 1 µL each of the forward and reverse primers per sample were accurately added to the PCR tube, resulting in a total volume of 25 µL. Following this, the tubes underwent centrifugation at maximum speed and were then placed in the PCR device. The temperatures for the three PCR stages were set as follows: 95 °C for 15 s, 56 °C for 30 s, and 72 °C for 30 s. These stages were repeated for 40 cycles.

### 2.5. Statistical Analysis

Statistical analysis was conducted utilizing SPSS version 22. Variations in proportions were assessed using the Chi-square test. A significance level of *p* < 0.05 was chosen to determine statistical significance.

## 3. Results

### 3.1. Clinicopathological Features within the Study Population

The clinicopathological characteristics of the patient’s population, including estrogen receptor (ER), progesterone receptor (PR), and human epidermal growth factor receptor 2 (HER2), are summarized in Table 1. The final analysis comprised 110 tumor samples (10 samples were excluded from the analysis due to the unavailability of their molecular subtyping data), with the mean age of 55.7 years, and a standard deviation (SD) of ±11.6. There was a slightly larger number of patients under 56 years of age (n = 56, 51%) than patients older than 56 years of age (n = 54, 49%). Molecular subtyping of BC revealed that 66% were luminal A (ER+ and/or PR+ with HER2−), 19% were luminal B (ER+ and/or PR+ with HER2+), 6% were HER2+ positive only (ER and PR both negative), and 9% were triple-negative BC (TNBC). Additionally, clinical staging of the sample cohort showed that 44% were classified as stage I, 16% as stage II, 14% as stage III, and 26% as stage IV. Lymphovascular invasion was identified in over half of the samples (52%; 57/110), while lymph node involvement by cancerous cells was observed in 67% (74/110) of cases. These clinicopathological characteristics were selected because BC is characterized by its heterogeneity, encompassing various histological subtypes with unique clinicopathological profiles. The prognosis for these special histological types varies significantly, indicating profound heterogeneity within the disease. Importantly, the histological subtype serves as an independent prognostic indicator, emphasizing its crucial role in predicting outcomes for BC patients.

### 3.2. Detection of High-Risk HPV Subtypes, EBV, and CMV by RT-PCR

Our data show that 27 out of 110 samples tested positive for high-risk HPVs (HR-HPVs), accounting for 24.5% of the total sample number (Table 1). Among those, the most frequently detected HR-HPV subtype was HPV16, which was present in 23 (85.2%) cases, followed by HPV18 (n = 3, 11.1%), and other genotypes (one of the following HR-HPV genotypes 31, 33, 35, 39, 45, 51, 52, 56, 58, 59, 66, 68) seen in 3 (11.1%) samples. HPV double infection (HPV16 and others) was seen in two samples (7.4%) (Table 1).

**Figure 1:**
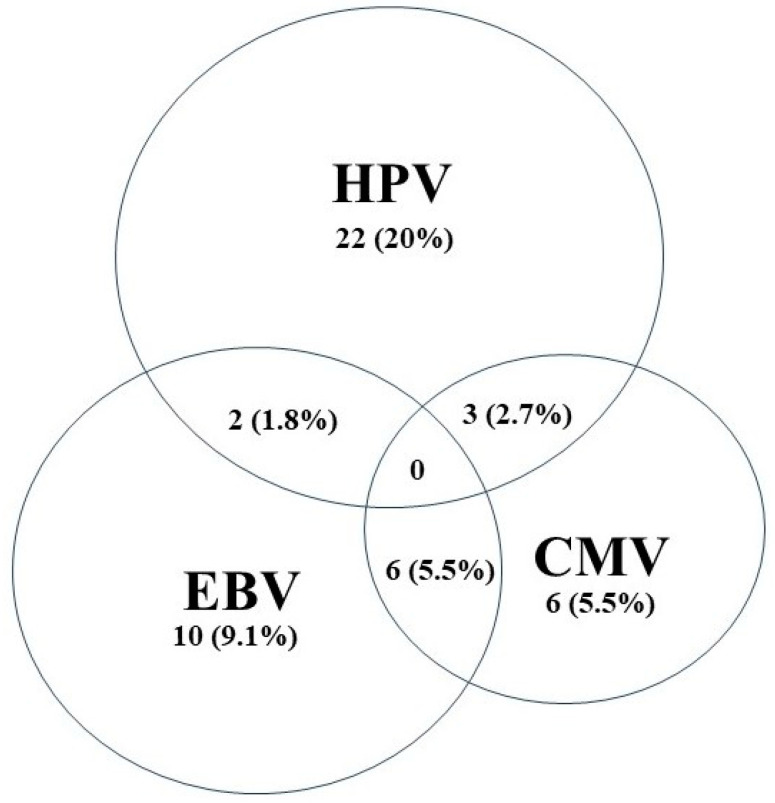
Venn diagram depicting single and multiple infections with HPV, CMV, and EBV in Jordanian breast cancer samples (n = 110).

On the other hand, we observed that 18 samples (16.4%) tested positive for EBV and 15 samples (13.6%) tested positive for CMV. More notably, our data show that HR-HPVs and EBV coinfection occurred in 1.8% (n = 2), HR-HPVs and CMV coinfection occurred in 2.7% (n = 3), and EBV and CMV coinfection occurred in 5.5% (n = 6) of the investigated BC cases (Figure 1).

Lastly, none of the normal breast samples showed positivity for HR-HPVs or CMV, with only one sample being positive for EBV. These variations among normal breast samples were statistically insignificant (*p* > 0.05).

### 3.3. Clinicopathological Association with HR-HPV, CMV, and EBV Positivity

An analytical association was conducted between each viral infection and the clinicopathological characteristics of the 110 tumor samples (Table 1). Among individuals aged ≤56 years, HPV was the most frequently observed, with 18 positive cases compared to 7 for CMV and 8 for EBV. Conversely, within the age group >56 years, an almost similar number of positive cases were found for each viral marker, with 9 for HPV, 8 for CMV, and 10 for EBV.

Among luminal A tumors, 20 were positive for HPV, which was the highest among the three screened viruses, compared to 10 and 11 positive cases for CMV and EBV, respectively. Similar findings were observed in luminal B cases, where HPV positivity was highest with four cases compared to two cases for each CMV and EBV. Among HER2+ cases, two were positive for HPV, while only one sample was positive for CMV or EBV. Notably, only one case of TNBC was positive for HPV, while two cases were positive for CMV and four for EBV.

Regarding clinical stage, 10 stage I cases exhibited positive HPV compared to 11 and 9 for CMV and EBV, respectively. In stage II samples, six were HPV positive, two for EBV, and no positive cases were observed for CMV. Among stage III tumors, there were four samples with positive HPV and three cases positive for CMV or EBV. In stage IV samples, HPV positivity was observed in seven cases, while only one case was positive for CMV and four for EBV.

HPV positivity was reported in 15 cases with lymphovascular invasion, whereas only 4 and 7 cases were reported positive for CMV and EBV, respectively. In contrast, among lymphovascular-invasion-free samples positivity rates were 12, 11, and 11 cases for HPV, CMV, and EBV, respectively. Among the subset where lymph nodes were involved, there were 18 cases positive for HPV, 7 for CMV, and 12 for EBV. Conversely, in cases that lacked lymph nodes involvement, nine were positive for HPV, seven for CMV, and five for EBV.

Out of the 110 tumor samples analyzed, 14 patients had a history of smoking. Among these, four patients tested positive for HPV, four patients tested positive for CMV, while only one patient tested positive for EBV. Conversely, among the 96 patients with no previous smoking history, 23, 11, and 17 tested positive for HPV, CMV, and EBV, respectively (Table 1).

Additionally, we investigated the relationship between family history and the presence of HPV, CMV, and EBV. We observed that the majority of HPV-positive cases (22 out of 27) had a positive family history of breast cancer. Conversely, most of the CMV-positive cases (17 out of 21) and EBV-positive cases (11 out of 18) were found in patients with no previous family history of breast cancer (Table 1).

Regarding the correlation between carcinoma in situ (CIS) and viral infections, we found that most cases were seen in patients lacking all three viral infections: 56 out of 83 in HPV-negative cases, 69 out of 95 for CMV-negative cases, and 70 out of 92 in EBV-negative cases. For tumor size correlation, 16 out of 27 HPV-positive cases, 7 out of 15 CMV-positive cases, and 11 out of 18 EBV-positive cases had tumors with dimensions exceeding 3 cm (Table 1).

Finally, we explored the relationship between HPV, CMV, EBV, and distant metastasis. The majority of samples positive for viral infections were observed among patients without distant metastasis (Table 1).

It is notable that a statistically significant association (*p* < 0.05) was observed between HPV status and age (*p* = 0.047), indicating that most HR-HPV infections (66.6%) occurred among younger aged patients. Clinical staging and CMV infection were also statistically significant (*p* = 0.015), highlighting that most CMV infections (73.3%) occurred in patients diagnosed with stage I BC (Table 1). Despite lacking statistical significance, it was noted that most cases of HR-HPV, CMV, and EBV infections were recorded in patients with grade II BC. CIS and HPV infection showed a statistically significant correlation indicating that CIS in BC is not necessarily linked to HPV infection or arises as a consequence of it, as observed in cervical cancer.

## 4. Discussion

This study represents the first investigation into the presence/co-presence of HR-HPVs, CMV, and EBV in human BC and its association with the immunohistochemical subtyping within a Jordanian subpopulation. Our findings indicate that HR-HPVs were detected in 24.5% of BC samples, whereas none of the normal breast samples tested positive for HR-HPVs. Previous studies have reported the presence of HR-HPVs in BC patients worldwide, including in samples from the Middle East and North Africa (MENA) region, with prevalence ranging from 21% to 65% [28,29,30,31]. Specifically, our results align with several reports from Egypt, Iran, and Jordan, where high rates of HR-HPVs (22.2%, 25.9%, and 21%, respectively) were observed [28,32,33]. However, it is noteworthy that in the Jordanian population, HPV prevalence seems to largely vary (range: 31–75%) in different types of cancers, including those closely associated with its infection such as oropharyngeal and cervical cancers, with HPV 16 being the most prominent [17,34]. HPV 16 genotype was the most predominant genotype in BC among Jordanian women in our study, similar to the Iraqi and Egyptian population [32,35]. HPV 18 genotype was reported as the most common in BC patients in Iran [29,33], and HPV 52 genotype among the Qatari and Lebanese populations [30,31]. Interestingly, the sole study examining the prevalence of BC among Jordanian women, conducted in 2020 by Al Hamad et al., reported HPV 18 as the most prevalent genotype in their studied subpopulation [28]. However, our findings are more consistent with all previous studies conducted in Jordan, which have collectively identified HPV 16 as the most predominant genotype among patients with cervical and oropharyngeal cancers [17,18,36,37,38].

Our findings are consistent with HPV genotypes that are commonly found in cancers worldwide where most cases are caused by genotypes 16 and 18. Likewise, a Canadian study revealed HPV 16 genotype as the most frequent in BC among Canadian women, whereas BC samples from the Qatari population did not detect the presence of HPV 16 [31,39]. Additionally, PCR analysis on BC samples from Syria detected HPV 33 genotype as the most prevalent subtype, followed by HPV genotypes 35, 18, and 16 [40]. Overall, the variation in HPV prevalence and genotype distribution can be attributed to different geographical distribution, sample size, and methodological differences. Among our cohort, only one patient infected with HPV was diagnosed with colon cancer and mucoepidermoid carcinoma of the parotid gland. Despite multiple studies showing relation between cervical lesions and the presence of HPV in BC, none of the patients with HPV infection in our study were diagnosed with cervical or oropharyngeal cancers [13].

Studies investigating the presence of CMV in BC patients in the MENA region are scarce. Worldwide, the detection of CMV among BC patients ranged from 0–100%. In the MENA region six studies were found: three in Iran, two in Egypt, and one in Iraq. In these studies, CMV was detected in 6.3–54% of the BC tissue compared to 0–28% in controls [41,42,43,44,45]. In our study, CMV was detected in 13.6% of the patients, which aligns with previous studies [42,43,44,46]. It is noteworthy that a New Zealand study revealed that CMV was completely absent in BC tissue samples from New Zealand women [47], while a study in China has shown that all BC cases were positive for CMV [48]. The differences in CMV distribution can be attributed to age, different socioeconomic status, hygiene practices, sexual behaviors, immune status, healthcare infrastructure, and geographical variations [49,50,51,52]. Table 2 summarizes the prevalence of CMV in BC tissue and controls among patients in the designated countries.

EBV presence is detected in approximately 30–50% of cancer cases globally. Studies from Turkey and Syria have reported EBV positivity rates of 58% and 52%, respectively, in BC cases, while reports from Qatar, Tunisia, Lebanon, and Egypt revealed EBV presence in 49%, 44%, 40%, and 37.5% of BC samples, respectively [23,30,31,53,54,55]. Additionally, EBV presence in BC tissue has been reported in Iran and Iraq in 27%, and 22.5%, respectively [56,57]. In our study, we found that EBV is present in approximately 16.4% of BC cases in Jordanian women. However, the mechanism by which EBV infects mammary epithelial cells is still not fully understood. A study by Hu et al. demonstrated that EBV infects mammary epithelial cells expressing CD21 and promotes the growth of early mammary epithelial cells with a stem cell phenotype [58]. EBV infection alters gene expression and stimulates oncogenic signaling via c-MET [58]. Moreover, EBV infection, along with activated RAS, triggers BC development; however, EBV is not essential once malignant transformation has occurred [58]. Our data suggest that the prevalence of EBV in BC tissues in Jordan is lower than its prevalence in the MENA and the world.

**Table 2:** Prevalence of CMV in BC tissue and controls among patients in the indicated countries.

Country	Year	CMV Positive in BC	CMV Positive in Controls	Reference
New Zealand	2015	0%	3%	Richardson et al. [47]
Egypt	2018	18%	5%	El Shazly et al. [43]
China	2018	100%	---	Cui et al. [48]
Iraq	2018	6.3%	5%	Kadhim et al. [56]
Iran	2019	54%	28%	Sepahvand et al. [45]
Iran	2021	33%	12%	Fard et al. [41]
Iran	2021	16.3%	2%	Nakhaie et al. [42]
Canada	2022	18.4%	10%	Yang et al. [46]
Egypt	2024	13.8%	0%	Elnegery et al. [44]

Previous studies indicate that the co-occurrence of HR-HPVs and EBV contributes to the onset and advancement of various cancers, including BC [59,60]. Our findings indicate that only 1.8% of BC cases in Jordanian women are co-infected with both high-risk HPVs and EBV. This contrasts with studies conducted in Qatar, Syria, and Lebanon, where high-risk HPVs and EBV were co-present in 47%, 32%, and 29% of BC samples, respectively [30,31,61]. Additionally, coinfection of high-risk HPVs and CMV was observed in 2.7% of cases, and EBV and CMV coinfection in 5.5% of BC cases. These results support the potential cooperative role of HR-HPVs and EBV oncoproteins in initiating and/or advancing various subtypes of human BC, as reported previously [31,54,62,63].

## 5. Conclusions

In conclusion, our findings highlight the predominance of HR-HPV infection among a Jordanian female subpopulation, and the co-existence of HR-HPV, CMV, and EBV coinfections suggesting an association with tumor grade and stage. This investigation proposes that an HPV vaccine could potentially be utilized to prevent the development and progression of specific subtypes of BC, yet the oncogenic role of HPV in BC requires further analysis. Moreover, future research involving larger cohorts in Jordan and the MENA region to validate the co-presence and HPV subtypes in BC is welcomed.

## Figures and Tables

**Table 1:** Clinicopathological association of HR-HPV, CMV, and EBV status among malignant breast samples (n = 110). HR-HPV: high-risk human papilloma virus; CMV: cytomegalovirus; EBV: Epstein–Barr virus; ER: estrogen receptor; PR: progesterone receptor; HER2: human epidermal growth factor receptor 2, LV: lymphovascular invasion; LN: lymph node involvement; CIS: carcinoma in situ; +: positive; −: negative. Bolded *p* values indicate statistical significance (*p* < 0.05).

		HR-HPV			CMV			EBV		
		+	−	*p* Value	+	−	*p* Value	+	−	*p* Value
Age	≤56	18	38	**0.047**	7	49	0.786	8	48	0.612
	>56	9	45		8	46		10	44	
Luminal A	ER+/PR+, HER2−	20	53	0.595	10	63	0.783	11	62	0.166
Luminal B	ER+/PR+, HER2+	4	17		2	19		2	19	
HER+	ER−/PR−, HER2+	2	4		1	5		1	5	
TNBC	ER−/PR−, HER2−	1	9		2	8		4	6	
Stage	Stage I	10	38	0.726	11	37	**0.015**	9	39	0.841
	Stage II	6	12		0	18		2	16	
	Stage III	4	11		3	12		3	12	
	Stage IV	7	22		1	28		4	25	
Grade	Grade 1	0	10	0.110	3	7	0.147	2	8	0.798
	Grade 2	18	42		9	51		9	51	
	Grade 3	9	31		3	37		7	33	
LV	Present	15	42	0.666	4	53	0.051	7	50	0.304
	Not identified	12	41		11	42		11	42	
LN	Positive	18	56	0.454	7	67	0.131	12	62	1.000
	Negative	9	22		7	24		5	26	
Smoking history	Yes	4	10	0.743	4	10	0.098	1	13	0.459
	No	23	73		11	85		17	79	
Family history	Positive	22	64	0.238	3	11	0.511	7	13	0.057
	Negative	3	17		17	75		11	75	
CIS	Positive	2	56	0.011	12	69	0.755	11	70	0.242
	Negative	25	27		3	26		7	22	
Tumor size	>3 cm	16	39	0.376	7	78	1.000	11	44	0.440
	≤3 cm	11	44		8	47		7	48	

## Data Availability

The data presented in this study are available from corresponding authors upon request.
